# Supplementary material for: Interpreting the socio-technical interactions within a wind damage–artificial neural network model for community resilience
Source: R Soc Open Sci. 2020 Nov 18;7(11):200922. doi: 10.1098/rsos.200922 (PMC7735332; doi:10.1098/rsos.200922)
Supplement: Interpreting Socio-Technical Interactions in a Wind Damage ANN [file rsos200922supp1.docx]

**SUPPLEMENTARY INFORMATION**

**Interpreting the Socio-Technical Interactions within a Wind Damage-Artificial Neural Network Model for Community Resilience**

Stephanie F. Pilkington and Hussam N. Mahmoud

The information provided and discussed within this document is intended to serve as additional detailed information concerning data and methods used in support of the main article “Interpreting a Wind Damage Artificial Neural Network using Graph Theory. Note that within this Supplementary Information, Model (A) was originally Damage Model 3 and Model (B) was originally Damage Model 8 within the comparison analysis of 10 different ANN Models discussed herein.

**S1. Data Collection**

In order to build an ANN that could be used to ultimately model the damage and subsequent recovery from a wind hazard scenario, historical events were evaluated for relevant information-based data availability. Within this study, the National Weather Service Damage Survey viewer was used to analyze damage photos for the structural characteristics and their respective damage states following a wind-related weather even. This site also listed a wind speed associated with each geo-tagged damage photo. These photos and satellite images provided the structural and hazard related inputs, respectively, as introduced in Supplementary Table S1. These inputs are also the variables typically considered when determining and modeling building damage. The remaining data was extracted from the 2015 U.S. Census American Community Surveys (ACS). Supplementary Table S1 lists each of these variables and which models contain what input variables with Supplementary Table S1-A providing additional variable details. The variables listed Supplementary Table S1-A were chosen based on factors used to create structural fragilities, common structural concerns found in case studies, social vulnerability related to natural hazards, and any other attributes of the hazard that would ultimately affect the structure. Some of these parameters, such as percent area forested and the size of the hazard event, were intended as options in communicating potential debris impacts of a building, to an ANN learning system. In other words, increased number of trees nearby indicates a potential for branches to become projectiles, while a larger tornado event would encompass more debris to be transported across the path. Similar logic was used in deciding to incorporate the housing density, since damaged buildings also create debris. All models contain the hazard and structural input variables. The model variations serve to evaluate which social vulnerability factors can be linked to building damage as well.

**Supplementary Table S1.** List of variables and associated models that contain each. (*denotes variable considered in the current physics-based modeling approaches).

| Input Category | Variables | Models Associated |
| --- | --- | --- |
| Hazard | Wind type, speed*, and event size | 1, 2, 3, 4, 5, 6, 7, 8, 9, 10 |
| Structural* | Year built, occupancy, roof & wall materials, roof shape, footprint area. | 1, 2, 3, 4, 5, 6, 7, 8, 9, 10 |
| Surface Roughness* |  | 1, 2, 3, 4, 5, 6, 7, 8, 9, 10 |
| Surface Vegetation | Estimated percent forested and impervious surfaces | 1, 7, 8, 9, 10 |
| Tenure | Percent own and rent | 1, 2, 8, 9, 10 |
| Densities | Housing density, population density | 1, 7, 8 |
| Total Population |  | 1, 2, 4, 5, 6, 7, 8, 9, 10 |
| Median Age |  | 1, 2, 6 |
| Race | Percent Asian, African American, Native American, and Hispanic | 1, 2, 6 |
| Industry Employment | Extractive and service industries | 1, 2, 4 |
| Income | Per capita and the income-to-poverty ratio | 1, 2, 4, 9, 10 |

**Supplementary Table S1-A.** Variable details and data source.

| **Input Category** | **Input Sub-Category** | **Description** | **Data in numerical form?** | **Source** |
| --- | --- | --- | --- | --- |
| Hazard | Wind Speed | Estimated wind speed from damage assessment | Y | National Weather Service Damage Survey Viewer. |
|  | Wind Type | Straight-line winds or tornadic winds | N |  |
|  | Event Size | Area covered by storm | N |  |
| Locational | Surface Roughness | ASCE designations based on surrounding landscape from images and satellite. | N | American Society of Civil Engineers |
|  | % Impervious Surfaces | Approximately how much of the surrounding area consists of roads, buildings, or other non-natural surfaces. | Y | Google Satellite view |
|  | % Forested | Of the remaining pervious surfaces, approximated percent area with trees. | Y |  |
|  | Housing Density | Number of housing units per area | Y |  |
|  | Median Age |  | Y | U.S. Census |
|  | % African American | Percent of total population | Y |  |
|  | % Hispanic |  | Y |  |
|  | % Native American |  | Y |  |
|  | % Asian |  | Y |  |
|  | % Own | Percent owning their own property (tenure) | Y |  |
|  | % Rent | Percent renting their housing (tenure) | Y |  |
|  | Per Capita Income |  | Y |  |
|  | Income:Poverty | Income to Poverty ratio | Y |  |
| Buildings | Year Built | Median year built for census block | Y |  |
|  | Occupancy | IBC Occupancy Class | N | International Building Code |

**Supplementary Table S1-A.** Variable details and data source (Continued).

| **Input Category** | **Input Sub-Category** | **Description** | **Data in numerical form?** | **Source** |
| --- | --- | --- | --- | --- |
| Buildings | Wall Material | The actual MWRFS and the façade. Connections were assumed to be based on the materials. | N | Visual: NWS Damage Survey Viewer photos. |
|  | Roofing Materials | The actual MWRFS and the cover. Connections were assumed to be based on the materials. | N |  |
|  | Roof type/shape | Standard roof designations | N |  |
|  | Extra Measures | If a note of structural measures such as hurricane straps/ties. | N |  |
|  | Height | The equivalent number of stories. | Y |  |
|  | Footprint Area | Estimated as very small (trailer) to extra-large (shopping mall). | N |  |

NWS survey photos from January 1, 2011 to December 31, 2015 were used to match data availability of ACS block group demographics. However, it is worth noting that ACS data does have significant error distributions and will therefore impact the resulting ANNs built. ACS data was available by census block, block group, tract, and county. For this research it was determined that an individual building would be located within a census block group and would assume the characteristics of that group. The U.S. Census block groups were chosen for the social parameters because, while it is the second smallest size, it is not too fine as to create issues in processing large data sets. Census block groups, like blocks, are bounded by roads, water, and governmental defined boundaries and are therefore different shapes and sizes. The relevant demographic data of a block group assigned to a building spatially residing within its boundaries, were chosen based on peer reviewed vulnerability indices. This data was considered as the building’s surrounding conditions potentially affecting the structure itself.

With an ANN, all data must be in numeric form; therefore, a key was created to denote structural component type designation (such as roofing material), hazard type (such as tornadic versus straight-line winds), and locational characteristics (such as surface roughness). This full key is provided below in Supplementary Tables S2 and S3. While Census data (social vulnerability) represented a numerical range (i.e. low income to high income values), the structural data was mostly categorical as observed from images. However, in reconciling this discrepancy, the categories in Supplementary Table S3 were established with some sense of order. For example, the materials were “ranked” by their modulus of elasticity such that a lower ID value of “1” would indicate a material with a lower modulus of elasticity than the other building materials.

**Supplementary Table S2.** Building code (surface roughness and occupancy) key for ANN inputs.

| **Parameter** | **Code Notation** | **Brief Description** | **ANN Notation** |
| --- | --- | --- | --- |
| ***Surface Roughness Categories*** | B | Urban and suburban areas, wooded areas, or other terrain with numerous closely spaced obstructions having the size of single-family dwellings or larger. | 2 |
|  | C | Open terrain with scattered obstructions having heights generally less than 30 ft (9.1 m). This category includes flat open country, grasslands, and all water surfaces in hurricane prone regions. | 3 |
|  | D | Flat, unobstructed areas and water surfaces outside hurricane prone regions. This category includes smooth mud flats, salt flats, and unbroken ice. | 4 |
| ***IBC Use and Occupancy*** | | | |
| Assembly | A-1 | Assembly uses, usually with fixed seating, intended for the production and viewing of the performing arts or motion pictures. (Theaters, concert halls, TV studios admitting an audience) | 303.2 |
|  | A-2 | Assembly uses intended for food and/or drink consumption. (Restaurants, Bars, Banquet halls, casinos) | 303.3 |
|  | A-3 | Assembly uses intended for worship, recreation, or amusement, and other assembly not classified elsewhere in Group A. (Bowling alleys, community halls, gyms, lecture halls, libraries, museums, churches, billiards, waiting areas in transportation terminals). | 303.4 |
|  | A-4 | Viewing of indoor sporting events (tennis courts, swimming pools, arenas) | 303.5 |
|  | A-5 | Viewing of outdoor sporting events (stadiums, grandstands, amusement park structures) | 303.6 |
| Business | B | The use of building or structure or a portion thereof, for office, professional or service-type transactions, including storage of records and accounts. (Airport traffic control towers, animal hospitals, banks, salons, car wash, civic admin, clinic outpatient, educational occupancies for students above 12th grade, data processing, food processing, cafeterias < 2500 sqft, labs, post offices, professional services). | 304.1 |
| Education | E | The use of a building or structure or a portion thereof, by six or more persons at any one time for educational purposes through the 12th grade. Includes occupancy of more than five children 2.5 years of age or older who receive education or personal care for fewer than 24 hours per day (day care). | 305.1 |
| **Parameter** | **Code Notation** | **Brief Description** | **ANN Notation** |
| Factory | F-1 | Moderate hazard. (Aircraft appliances, athletic equipment, automobiles, bakeries, beverages over 16% alcohol, bicycles, boats, business machines, cameras and photo equipment, canvas, rugs, construction or agriculture machinery, disinfectants, dry cleaning, furniture, laundries, machinery, fabrics, television filming w no spectators, paper mills, film or printing, textiles, tobacco, wood) | 306.2 |
|  | F-2 | Low hazard. Industrial uses that involve the fabrication or manufacturing of non-combustible materials that during finishing, packing, or processing do not involve a significant fire hazard. (Beverages with < 16% alcohol, brick and masonry, foundries, glass products, gypsum, ice, fabrication and assembly of metal products) | 306.3 |
| High Hazard | H | The use of a building or structure, or a portion thereof, that involves the manufacturing, processing, generation or storage of materials that constitute a physical or health hazard in quantities in excess of those allowed in control areas complying maximum allowable quantity limits. |  |
|  | H-1 | Detonation hazard: Explosives, organic peroxide (Class UD), Oxidizer (class 4), Unstable reactive (Classes 4 & 3). | 307.3 |
|  | H-2 | Deflagration hazard or hazard from accelerated burning: Combustible dust, combustible liquid, cryogenic flammable, flammable gas, flammable liquid, organic peroxide (Class I), Oxidizer (Class 3), Pyrophoric, Water reactive (Class 3). | 307.4 |
|  | H-3 | Readily support combustion or pose a physical hazard: Combustible fiber, combustible liquid, consumer fireworks, cryogenic oxidizing, explosives (Division 1.4), flammable liquid, flammable liquid combination, flammable solid, organic peroxide (Class II & III), oxidizer (Class 2), Oxidizing gas, unstable reactive (Class 2), water reactive (class 2). | 307.5 |
|  | H-4 | Health hazards: corrosives, toxic materials | 307.6 |
|  | H-5 | Semiconductor fabrication facilities and comparable research and development areas in which hazardous production materials are used and the aggregate quantity of material is in excess. | 307.7 |
| Institutional | I-1 | Occupancy shall include buildings, structures, or portions thereof for more than 16 persons, excluding staff, who reside on a 24-hour basis in a supervised environment and receive custodial care. (Alcohol/drug centers, assisted living, care facilities, group homes, halfway houses) | 308.3 |
|  | I-2 | Occupancy shall include buildings and structures used for medical care on a 24-hour basis for more than five persons who are incapable of self-preservation. (Foster case, Detox facilities, hospitals, nursing homes, psychiatric hospitals). | 308.4 |
| **Parameter** | **Code Notation** | **Brief Description** | **ANN Notation** |
|  | I-3 | Occupancy shall include buildings and structures that are inhabited by more than five persons who are under restraint or security. (Correctional centers, detention centers, jails/prisons, reformatories). | 308.5 |
|  | I-4 | Day care facilities (more than five persons who receive custodial care for fewer than 24 hours per day). | 308.6 |
| Mercantile | M | The use of building or structure or portion thereof for the display and sale of merchandise, and involves stock goods, wares or merchandise incidental to such purposes and accessible to the public. (Department stores, markets, drug stores, motor fuel-dispensing facilities, retail stores, sales rooms) | 309.1 |
| Residential | R-1 | Occupancies containing sleeping units where the occupants are primarily transient in nature. (Hotels, boarding houses, congregate living facilities with more than 10 occupants) | 310.3 |
|  | R-2 | Occupancies containing sleeping units or more than two dwelling units where the occupants are primarily permanent in nature. (Apartment houses, non-transient boarding houses with more than 16 occupants, convents, dorms, frats and sororities, non-transient hotels, live/work units, monasteries, vacation timeshare properties) | 310.4 |
|  | R-3 | Houses. Occupancies where the occupants are primarily permanent in nature and not classifies as R-1, R-2, R-4, or I. (Buildings that do not contain more than two dwelling units, non-transient boarding houses with <16 occupants, transient boarding houses with <10 occupants, care facilities for <5 persons receiving care, lodging houses with <5 guest rooms) | 310.5 |
|  | R-4 | Occupancy shall include buildings, structures or portions thereof for more than five but not more than 16 persons, excluding staff, who reside on a 24-hour basis in a supervised residential environment and receive custodial care. (drug centers, congregate care facilities, group homes, halfway houses, social rehab facilities) | 310.6 |
| Storage | S-1 | Moderate hazard. Buildings occupied for storage uses that are not classified as S-2. (Aerosols, aircraft hangar, bags, bamboos, books and paper in rolls or packs, boots and shoes, indoor dry boat storage, glues, grains, clothing materials, sugar, tires, upholstery, wax candles) | 311.2 |
|  | S-2 | Low hazard. Buildings used for the storage of noncombustible materials such as products on wood pallets or in paper cartons with or without single thickness divisions or in paper wrapping. (Asbestos, beverages <16% alcohol, cement in bags, chalk and crayons, dairy products, dry cell batteries, electrical coils, empty cans, food products, glass, ivory, metals, parking garages, stoves, talc and soap stones, washers and dryers) | 311.3 |
| **Parameter** | **Code Notation** | **Brief Description** | **ANN Notation** |
| Utility and Miscellaneous | U | Buildings and structures of an accessory character and miscellaneous structures not classified in any specific occupancy. (Agriculture buildings, aircraft hangars, accessory to a one- or two- family residence, barns, carports, fences taller than 6 ft, silos, greenhouses, livestock shelters, private garages, retaining walls, sheds, stables, tanks, towers) | 312.1 |

**Supplementary Table S3.** Building materials and shapes key for ANN inputs.

| **Parameter** | | ***~ E* (GPa)** | **Example Descriptors** | **ANN Notation** |
| --- | --- | --- | --- | --- |
| ***Materials*** *(ordered by modulus of elasticity)* | Open (N/A) | 0 | Stadiums | 0 |
|  | Brick/ Clay | 2 | Brick veneer/façade, mortar connections. Typically, older buildings. Older culverts. | 1 |
|  | Asphalt | 3 | Roof shingles | 2 |
|  | PVC & Plastic | 3 | Water pipes & house siding | 3 |
|  | BUR | 4 | Built-Up Roofing | 4 |
|  | Wood/Timber | 11.3 | Wood studs, drywall, wood paneling, wood shingles. Typically, would not exceed 4 stories. | 5 |
|  | Unreinforced Masonry (URM) or Tilt-ups | 17 | Concrete blocks (CMU). Typically, older buildings. | 6 |
|  | Concrete/ Masonry (reinforced) | 30 | Poured concrete walls, basements, concrete blocks | 7 |
|  | Other Metals | 69 | Aluminum sheets, corrugated siding, metal connection brackets/nails. Typically, storage facilities. Some transmission towers. | 8 |
|  | Glass | 75 | Mainly windowed façade. | 9 |
|  | Steel | 200 | I-beams, gusset plates, roof joists, iron pipes, transmission towers. (use "89" after a decimal point) | 10 or 89 |
|  | Manufactured home |  | Trailers, mobile homes | 0.5 |
| ***Roof type/ shape*** *(ordered by peaks)* | Open | NA |  | 0 |
|  | Flat |  | Same story level | 1 |
|  |  |  | Various stories | 1.numberofstories |
|  | Domed |  | Single | 2 |
|  |  |  | Multiple | 2.numberofdomes |
|  | Stepped |  |  | 3 |
| **Parameter** | | ***~ E* (GPa)** | **Example Descriptors** | **ANN Notation** |
| ***Roof type/ shape*** *(ordered by peaks)* | Monoslope | NA | (theta <10) | 4.1 |
|  |  |  | (theta >10) | 4.2 |
|  | Sawtooth |  | Multi-Monoslope | 5.numberofslopes |
|  | Gable |  | (theta <7) | 6.1 |
|  |  |  | (theta >7) | 6.2 |
|  | Hip |  |  | 7 |
|  | Mansard |  |  | 8 |
|  | Gambrel |  |  | 9 |
|  | Intersecting (Gable) |  | Multiple (theta <10) | 10.1numberofgables |
|  |  |  | Multiple (theta >10) | 10.2numberof gables |
|  | Intersecting (hip) |  | Multiple | 11.numberofhips |
| ***Footprint Area*** | Extra-Small | NA | Shed, mobile home, equally small house | 1 |
|  | Small |  | Average size house, small office | 2 |
|  | Medium |  | Multi-person home, avg. size business office, small strip mall | 3 |
|  | Large |  | Mall, big box, industrial buildings | 4 |
|  | Extra-Large |  | Airports, stadiums | 5 |

Overall, if the data collected was already present in numerical form, such values were used for the respective variable inputs. However, if this was not the case, the numerical ANN notations created by the authors in the above tables were used as the variable inputs to the ANNs. These ANN notations were designed with the intent of flexibility among many different building characteristics. For example, some homes may consist of a wood frame (or Main Wind Force Resisting System (MWFRS) and a brick exterior or façade. In this case, the material can be combined numerically such that the façade is an additional decimal to the MWFRS notation; in this example a 5.1 numerical value would be entered as the ANN input for such a house. This was similarly true for roofs and the roofing material. An example of these designations is outlined in Supplementary Figure S1.

**
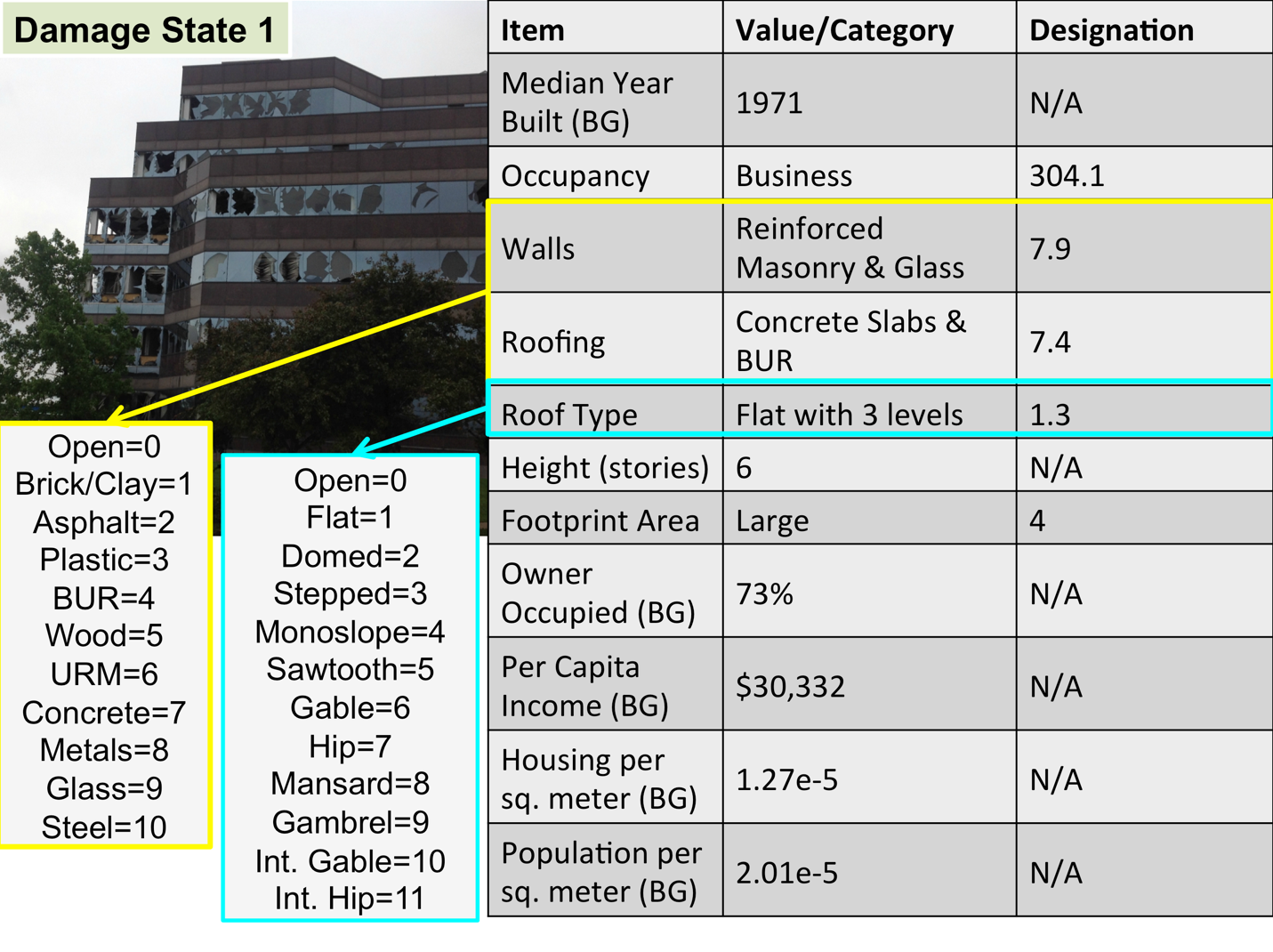
**

**Supplementary Figure S6.** Building data point with associated input variables (“Int.” = intersecting, “BG” = block group, and “BUR” = built up roofing; building damage image from NWS Damage Survey Viewer).

The ANN outputs were the building damage state given the provided input variables defined above. The output damage states were defined based on commonly accepted and previously applied definitions as outlined in Supplementary Table S4. In essence, each survey photo of a structure was assigned multiple attributes to illustrate its structural characteristics, surrounding demographics, hazard, and its ultimate damage state.

**Supplementary Table S4.** Windstorm Damage States for Buildings and EPN

| **Damage State** | **Range of damage ratio (%)** | **Description** | **Equivalent FEMA Assessment Description** | **Ex. NWS Wording or Image descriptions** |
| --- | --- | --- | --- | --- |
| 0 | 0 | No damage | No damage |  |
| 1 | > 0-10 | Slight damage | Affected. Some missing shingles. Cosmetic damage such as siding. | "Threshold of visible damage" (typically) |
| 2 | > 10 - 20 | Moderate damage | Minor. Nonstructural damage. Blown out windows. | "Loss of roof covering <20%" |
| 3 | > 25-50 | Substantial to heavy damage | Major. Failure or partial failure of structural elements. Missing roof but walls still intact. Water line 18" above floor. | "Large sections of roof structure removed." "Most walls remain." "Uplift of roof deck" |
| 4 | > 50 | Very heavy damage (destroyed) | Destroyed. Complete failure of at least two structural components. Imminent threat of collapse. | Exterior wall collapse, but some of roof might remain. |

In gathering this data, the most subjective, or difficult, portion was evaluating the photos found through the NWS Damage Survey Viewer. Only NWS damage points with photos could be used, but even those posed difficulties in deciphering the original structure shape and materials if the building was completely destroyed. If a photo showed a house (or even roof) completely gone, then the materials and roof shape were taken from context of nearby debris, neighboring homes, standard construction for the building type described, and prior satellite imagery where possible. Mixed materials, especially in the building façade, were taken into account through the use of decimal numbers and are further detailed in Appendix A. The occupancy type was typically noted in the damage survey, and if not, educated judgment was used based on structural appearance and the surrounding buildings. The ASCE surface roughness was typically assigned as either B or C depending on if the structure was in an urban or forested area, or in the plains. For example, farmlands were treated as surface roughness C. Examples of each of the damage states and some of the associated characteristics from photos throughout the state of Missouri for various extreme wind events are shown in Supplementary Figure S1 as well as Supplementary Figures S2 – S4.


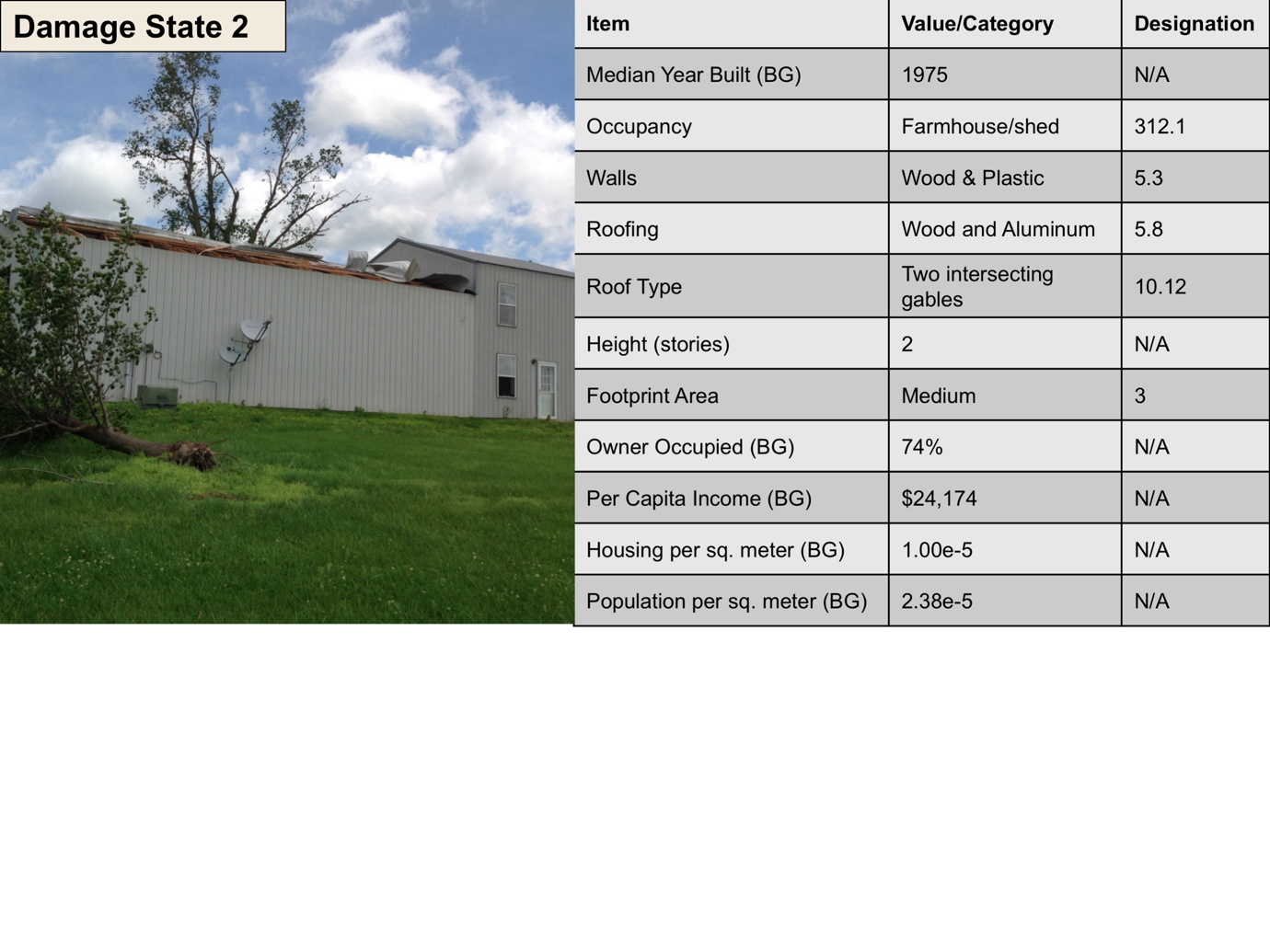


**Supplementary Figure S2.** Damage State 2 structure with example ANN input values.


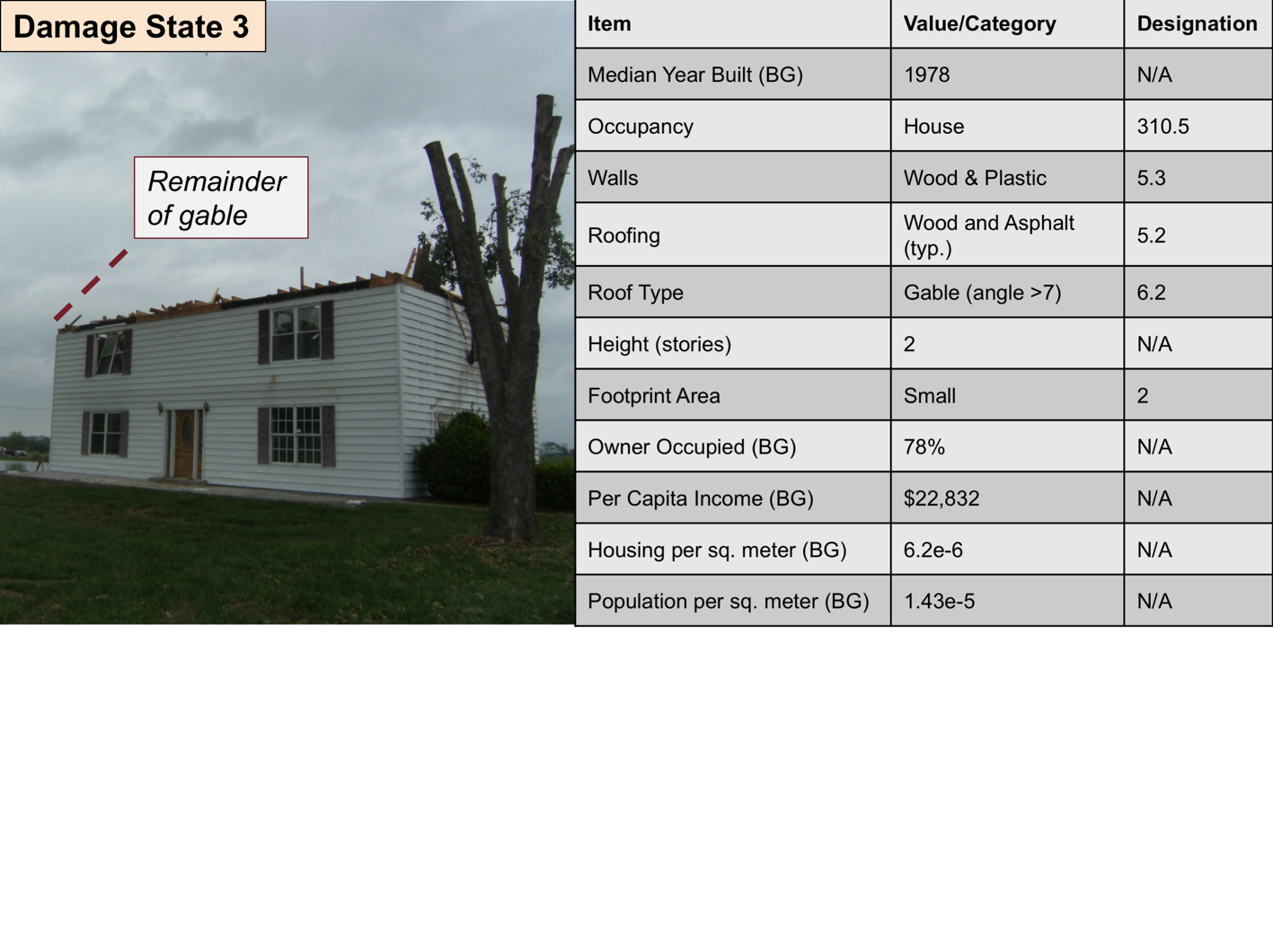


**Supplementary Figure S3.** Damage State 3 structure with example ANN input values.


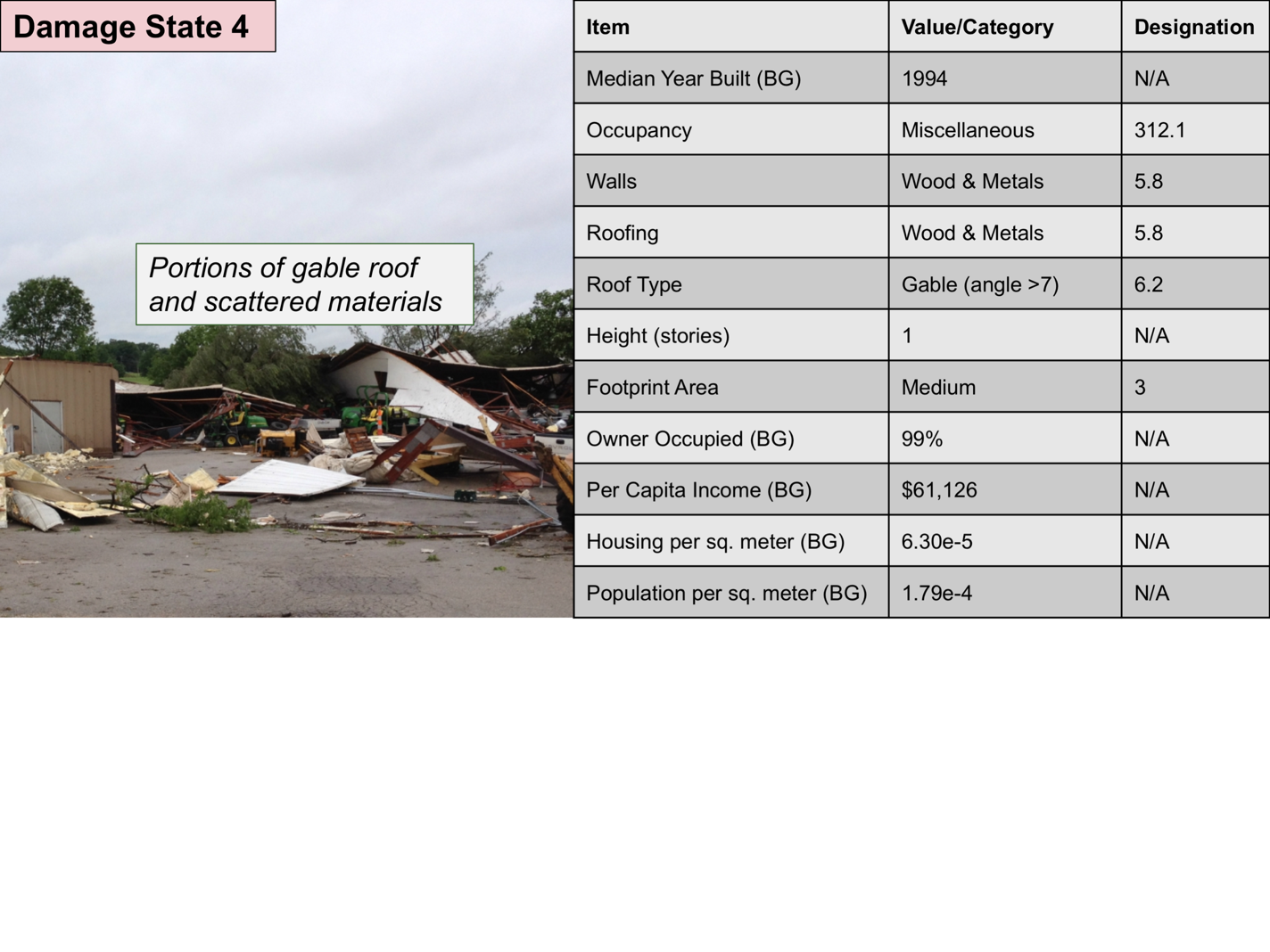


**Supplementary Figure S4.** Damage State 4 structure with example ANN input values.

The above figures are just 4 of the 117 data points used to build ANNs with varying training algorithms and for varying models (different input variables). The full data set is provided as a separate supplementary information file for this study.

**S2. Artificial Neural Network Variations**

The methods section following the main article outlines the ANN mathematical structure for Models (A) and (B), which, from this point on are now referred to as Damage Models 3 and 8, respectively. Supplementary Figure S5 reiterates the ANN structure being built and evaluated within this study, where *W* represents the weight from the *j*th to the *i*th neuron, *b* is the bias on the *i*th neuron, and *x* relates to the neuron values being “fed” into the following layer.


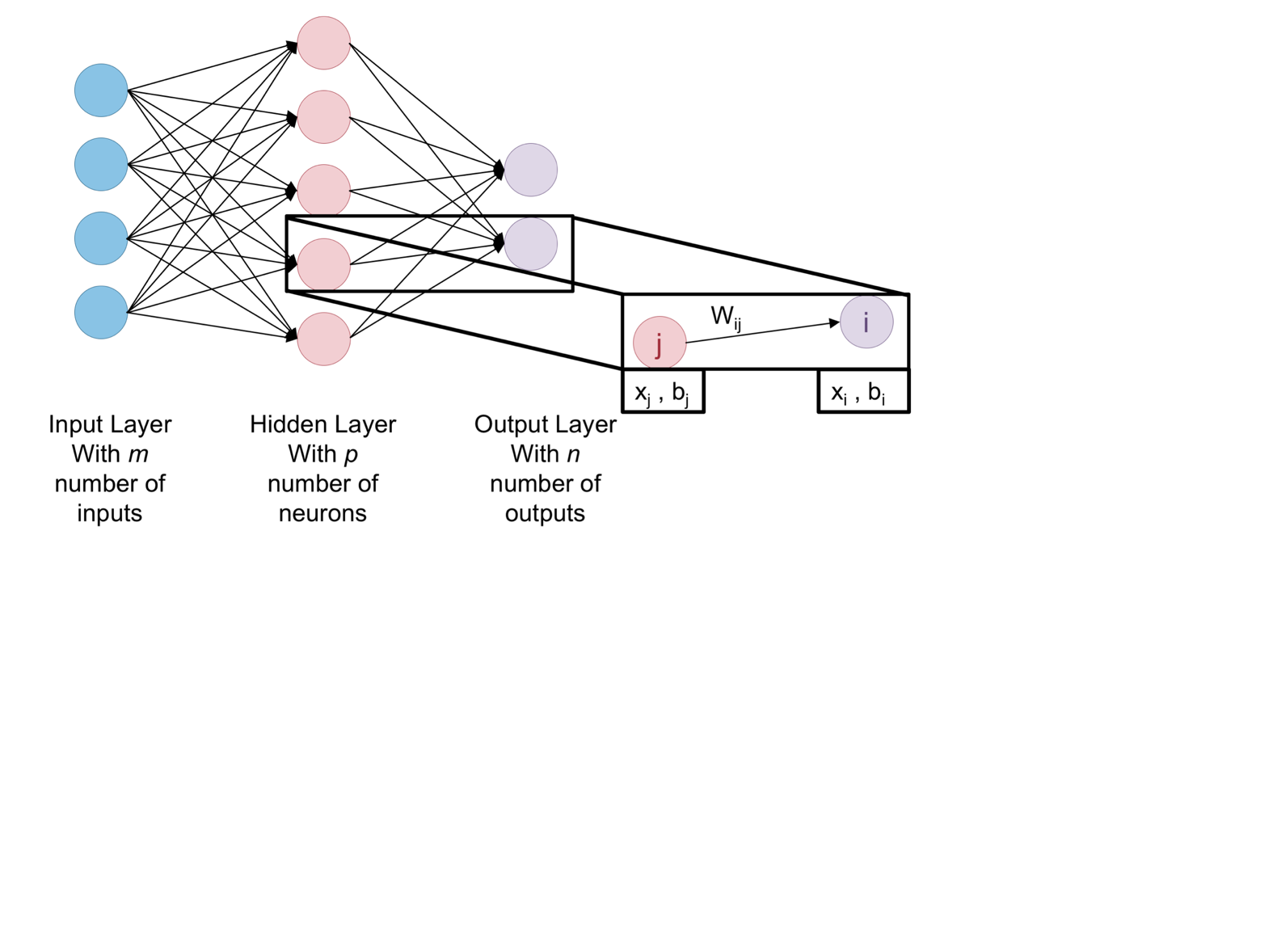


**Supplementary Figure S5.** Relationships of weights and biases between a network’s neurons.

However, before finalizing the two models within the main text, a training algorithm evaluation was conducted first. The target value, *Y*, for the ANNs was a function of the data set *D(x,y)* and the activation function, *s*. The error determined in Equation (8) of the Methods section was propagated back through the network to adjust the weights and biases as well as change the neuron activations by means of a myriad of training algorithm approaches. Most of these approaches predicate on first determining the gradient of the Cost (Error) Function (Equation 8) with respect to the weights ($-{\delta w}_{ij})$, such that

$\frac{\partial E}{{\partial w}_{ij}}=\frac{\partial E}{\partial y_{i}}\times\frac{\partial y_{i}}{\partial w_{ij}}=-\left( Y_{i}-y_{i} \right)x_{i}=-{\delta w}_{ij}$ (S-1)

The $-{\delta w}_{ij}$parameter would eventually update to a zero value in over-fitting cases if the log-sigmoid activation function was used in first normalizing the data instead of the tan-sigmoid function. Supplementary Table S5 outlines some of the training method variations used in updating the network’s weights and biases. Changing how the network learns, through each of these algorithmic methods, was one of the proposed variations to the ANNs in determining the best performing network for modeling building damage state from extreme wind events.

**Supplementary Table S5.** Learning Algorithm Variations for updating weights and biases.

| **Acronym** | **Name** | **Brief Description** |
| --- | --- | --- |
| LM | Levenberg-Marquardt | Least-squares curve fitting function. |
| RP | Resilient Backpropagation | Function fitting depending on the gradient of the transfer function with respect to the weights. |
| BFG | BFGS Quais-Newton | Function fitting with respect to the transfer function gradient. |
| SCG | Scaled Conjugate Gradient | Gradient descent with line search techniques to adjust “steps” to fit a function. |
| CGB | Conjugate Gradient with Powell/Beale Restarts | Function fitting with respect to the transfer function gradient. Update for each iteration is a factor of the previous gradient and the previous direction used to find the minimum point of the function. |
| CGF | Fletcher-Powell Conjugate Gradient | Function fitting with respect to the transfer function gradient. Update for each iteration is a factor of the square of the previous gradient and the square of the current gradient. |
| CGP | Polak-Ribiere Conjugate Gradient | Function fitting with respect to the transfer function gradient. Update for each iteration is a factor of the square of the previous gradient and current gradient. |
| OSS | One Step Secant | Function fitting with the goal of determining where the gradient is zero. |
| GDX | Variable Learning Rate Backpropagation | Function fitting similar to gradient descent with the incorporation of momentum training. |
| BR | Bayesian Regulation | Probabilistic approach using Bayes Theory. |

Within this research, the training algorithms and performance functions were first assessed for an initial model containing all the data gathered (Model 1) before moving on to evaluating model variations (Damage Models 1 through 10 with varying inputs). The training algorithms examined for Model 1 were defined in Supplementary Table S5. In evaluating the various training algorithms, the same performance function (MSE) was used followed by the SSE performance function. The mathematical differences between training functions are outlined as follows:

LM is a least-squares curve fitting function that began its first training iteration with an initial guess of *s* (the activation function) to be adjusted by $\delta$. As one of the first algorithms created for machine learning purposes, LM uses the Jacobian Matrix, ***J***, and identity matrix, ***I***, in order to adjust *s* by

$\left( J_{i}^{T}J_{i}+\lambda I \right)\delta_{ij}=\boldsymbol{J}_{\boldsymbol{i}}^{\boldsymbol{T}}\left[ y_{i}-f(s_{i}) \right]$ (S-2)

where $\lambda$ was considered a kind of “damping” variable that can change size for each iteration. Similarly, RP uses a weight related update value, *∆*, that ties to the weight value change, *∆w*, as follows:

$\Delta w_{ij}^{(t)}=\left\{ \begin{aligned} {-\Delta}_{ij}^{(t)} , if {\frac{\partial s}{\partial w}}^{(t)}>0 \\ {+\Delta}_{ij}^{(t)} , if {\frac{\partial s}{\partial w}}^{(t)}<0 \\ 0 , else \end{aligned} \right.$ (S-3)

with update-values following each iteration (*t*), as follows:

$\Delta_{ij}^{(t)}=\left\{ \begin{aligned} {\eta^{+}*\Delta}_{ij}^{\left( t-1 \right)} , if {\frac{\partial s}{\partial w}}^{\left( t-1 \right)}*{\frac{\partial s}{\partial w}}^{\left( t \right)}>0 \\ {\eta^{-}*\Delta}_{ij}^{\left( t-1 \right)} , if {\frac{\partial s}{\partial w}}^{\left( t-1 \right)}*{\frac{\partial s}{\partial w}}^{\left( t \right)}<0 \\ \Delta_{ij}^{\left( t-1 \right)} , else \end{aligned} where 0<\eta^{-}<1<\eta^{+} \right.$ (S-4)

These equations were executed for neuron bias values (*b*) much in the same way the weights were adjusted. Additionally, these procedures were performed for every possible neuron connection. In other words, **w** is a matrix containing all weights from the input to hidden layers and the hidden and output layers.

Similar to LM and RP, a significant portion of training algorithms were based in optimization using conjugate gradient where initial weight values were assumed and adjusted. This is to say that these algorithms were function-fitting problems that used a function gradient scale and direction to update weight (and bias) values. Simply put, this would describe weight updates such that:

$w_{ij}^{(t)}=w_{ij}^{(t-1)}+a\times\delta w_{ij}$ (S-5)

where *a* is the step to be minimized along the search direction $\delta w_{ij}$. Starting with a simpler version of how $\delta w_{ij}$ was determined, gives the conjugate gradient method in the form of the BFGS Quasi-Newton Learning Algorithm, which defines:

$\delta w_{ij}=\frac{-H}{{gw}_{ij}}$ (S-6)

where *gw_ij_* is the gradient at a specific point on the function and ***H*** is the Hessian matrix. This value was initiated as the steepest point then adjusted per the calculated network error. For the remaining algorithms, with the exception of BR, the determination of $\delta w_{ij}$ is outlined in Supplementary Table S6.

**Supplementary Table S6.** Conjugate Gradient Based Training Algorithms

| **Algorithm** | $\boldsymbol{\delta}\boldsymbol{w}_{\boldsymbol{ij}}\boldsymbol{=}$ | **where** | **Equation** |
| --- | --- | --- | --- |
| CGB | ${-gw}_{ij}+{\delta w}_{ij}^{(t-1)}$ |  | (S-7) |
| CGF | ${-gw}_{ij}+{\delta w}_{ij}^{(t-1)}\times\frac{r^{(t)}}{r^{(t-1)}}$ | *r* is the normal square of the gradient | (S-8) |
| CGP | ${-gw}_{ij}+{\delta w}_{ij}^{(t-1)}\times\frac{\left( {gw}_{ij}^{\left( t \right)}-{gw}_{ij}^{\left( t-1 \right)} \right)\times{gw}_{ij}^{\left( t \right)}}{r^{(t-1)}}$ |  | (S-9) |
| OSS | ${-gw}_{ij}^{\left( t \right)}+0.001\times{\delta w}_{ij}^{(t-1)}+0.1\times{gw}_{ij}^{\left( t-1 \right)}$ | The values 0.001 and 0.1 are used in this study but can be altered. | (S-10) |
| GDX | $MC\times{\delta w}_{ij}^{(t-1)}+LR\times MC\times\delta P/\delta w$ | *MC* is the momentum constant (=0.9), *LR* is the learning rate (0.01), and *P* is the performance of that iteration | (S-11) |

The majority of previously established learning algorithmic methods evaluated ANNs as a function-fitting problem. BR, however, is probabilistic focused in Bayes Theory such that,

$p\left( w_{ij} | D_{i} \right)=\frac{P\left( w_{ij} \right)P(D_{i}|w_{ij})}{\int P(w_{ij})P(D_{i}|w_{ij})}$ (S-12)
In other words, BR evaluates the probability of the weights, *w_ij_*, on the connections between neurons given the data set, *D*.

Within this research, the various training methods and square error types were explored for their impact on the resulting network built to address the problems of modeling building damage state from severe weather events. Following the network training method variations, different possible input arrangements were considered with the ultimate goal of evaluating what potential parameters should be used in determining impact and recovery of a community’s building stock.

Model variations for the inputs contributing to damage state were previously outlined in Supplementary Table S1, in which certain variables were lumped together, such as race (e.g. either all race categories were included in the model or none were). The 10 damage models were designed so that subsequent evaluation could determine if an ANN was able to capture debris potential and how some socioeconomic factors may influence the resulting damage states. The Model 1 algorithms and subsequent models of different inputs were evaluated using Performance Characterizing Indicators (PCIs) of network performance, percent error, TPR, TNR, FPR, and FNR. The model with the most desirable PCIs was then chosen as the final ANN for application and the one with the least desirable PCIs was chosen for comparison. However, in application, an ensemble consisting of multiple ANNs of the same structure (different weights and biases), for the best build performance across training algorithms and model input variations, was used for graphical analysis and in application.

**S3. Results of Artificial Neural Network Variations**

The training algorithms outlined in Supplementary Table S5 represent the core of current theories in neural network training. These theories utilize variations on gradient descent methods or Bayes Theory. The merit to applying a specific theory can change depending on the problem being modeled. Each ANN was “built” once the lowest possible error (MSE or SSE) had been reached through multiple iterations. Each ANN model herein was built a minimum of 50 times (with multiple iterations within each build) to determine a range and subsequent mean for each PCI for training algorithm capability comparisons.

In modeling building damage state, across most algorithms, the PCI’s were more desirable with the MSE performance function than the SSE function as shown in Supplementary Figures S6 through S8. These figures show the majority of builds that were within the +/-2SD by the thick bars and the maximum and minimums reached by the extended lines/nodes from those bars. The BR training algorithm (with a percent error mode of 4.3%) clearly best fits the desired PCIs out of all the training algorithm options with 100% of the builds bounded by the maximum and minimum values reached. The LM and RP training algorithms (percent error modes of 54.7% and 34.2%, respectively, for the MSE performance function) were the next best performing algorithms. While LM had better false negative rates, the percent error mode for RP was lower than LM. LM’s PCIs were also highly varied with a change from MSE to SSE performance along with the OSS algorithm approach. RP is the only algorithm that actually showed improved results when switched from the MSE to SSE performance. Note that when evaluating the performance functions, training performance is desired to be close to zero, which indicates that the ANN outputs are closely matching the targets.


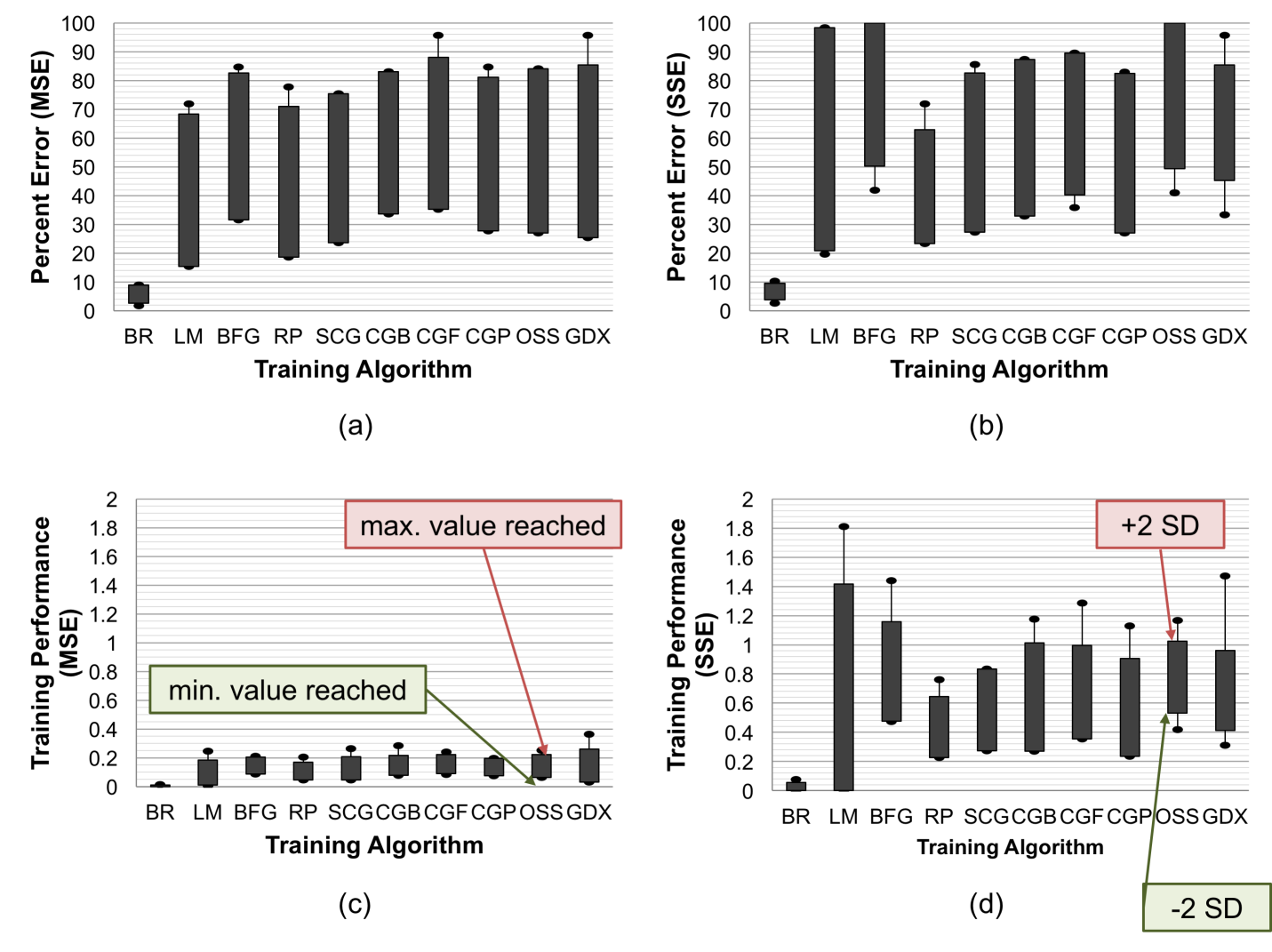


**Supplementary Figure S6.** (a) Percent error for MSE performance, (b) Percent error for SSE performance, (c) training performance for MSE performance and (d) training performance for SSE performance for the explored training algorithms.


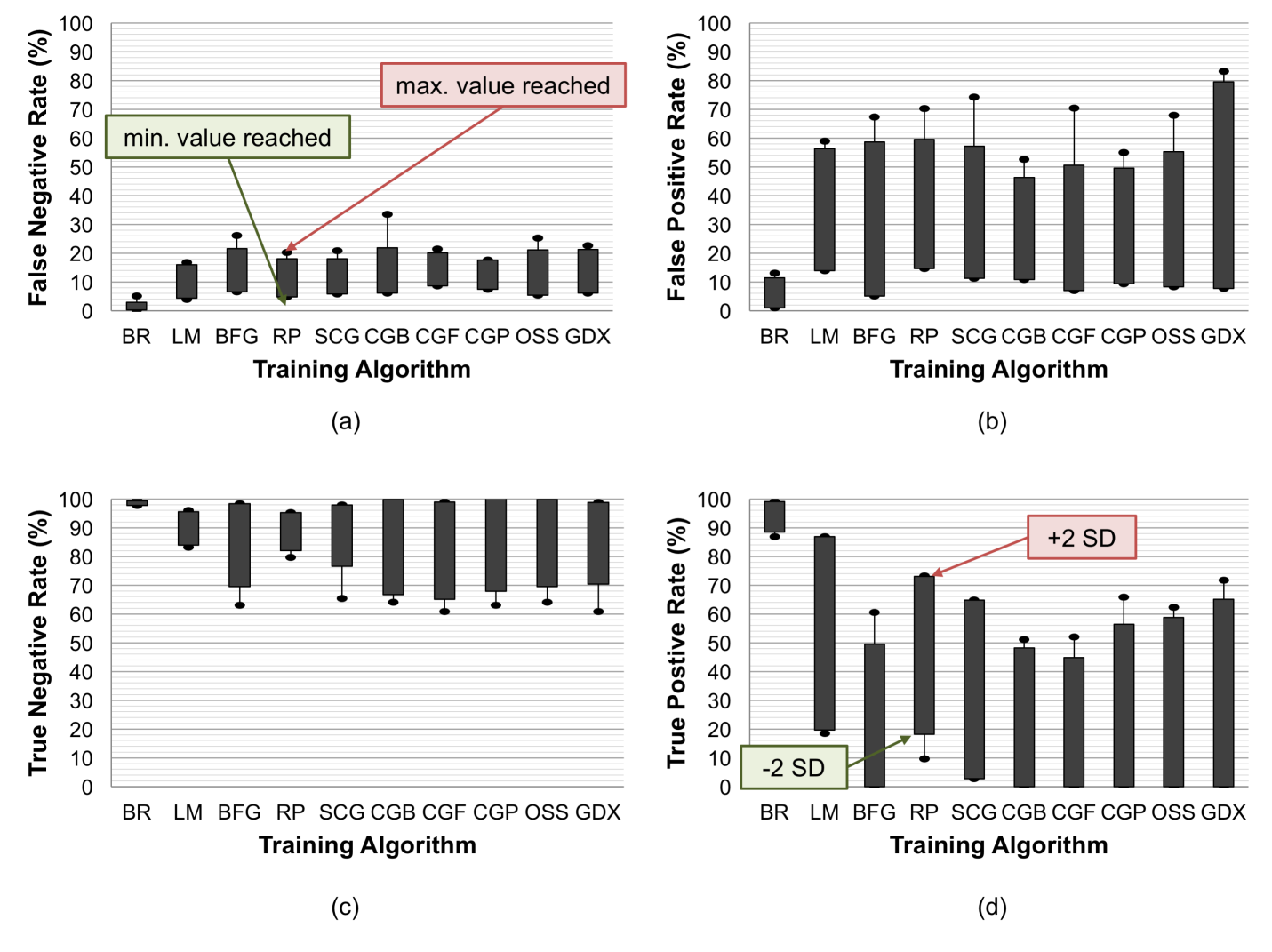


**Supplementary Figure S7.** Explored algorithms’ (a) FNR, (b) FPR, (c) TNR, and (d) TPR for MSE performance.


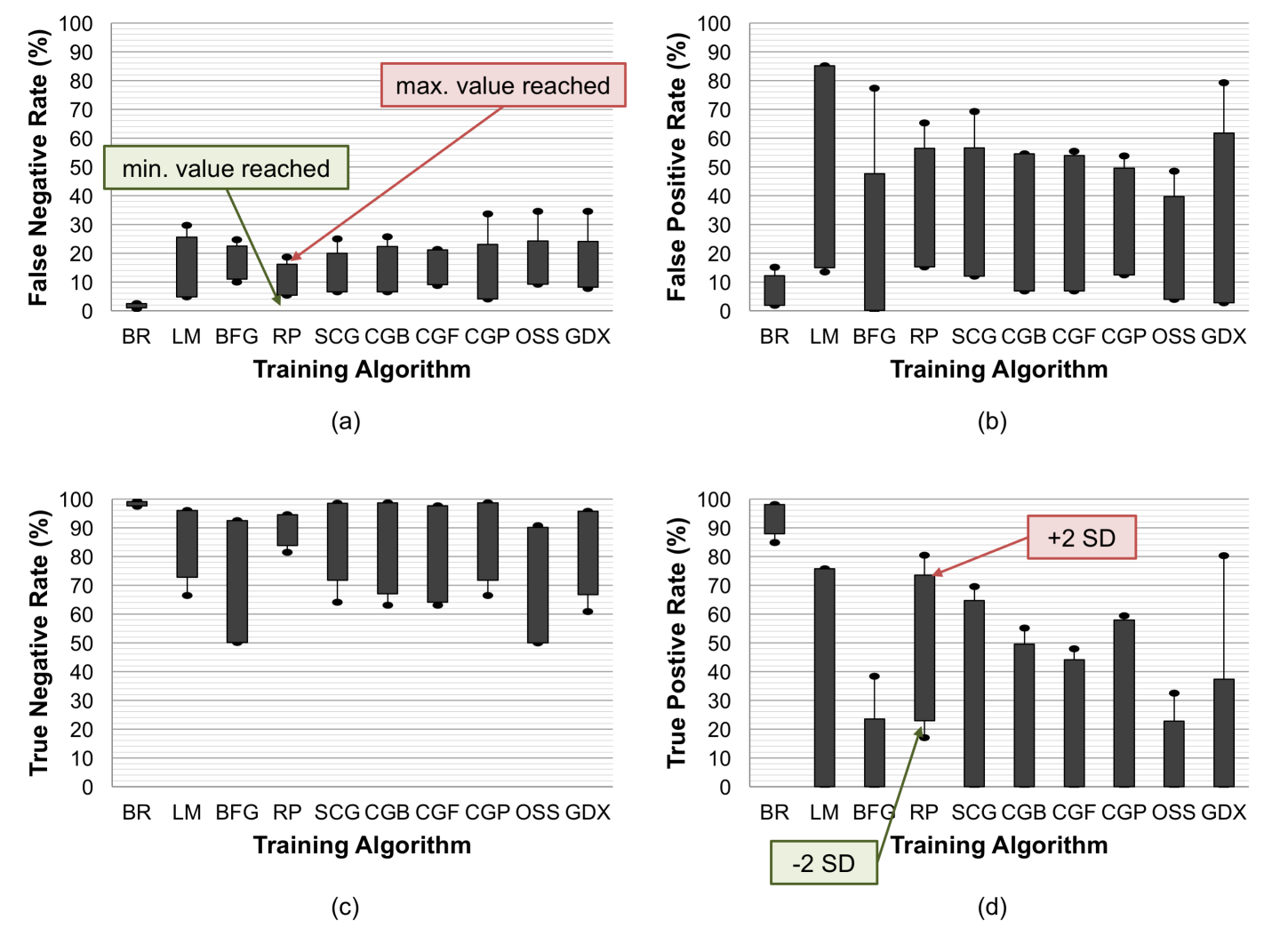


**Supplementary Figure S8.** Explored algorithms’ (a) FNR, (b) FPR, (c) TNR, and (d) TPR for SSE performance.

Based on the above results, BR with MSE performance, LM with MSE performance, and RP with SSE performance were chosen for the next step in evaluating model input variations to evaluate the effect of combining sociological and engineering related variables in determining damage state. While BR clearly produces the lowest percent errors in training, the LM and RP algorithms were also used in the following step as a check that the conclusions being drawn were consistent across each training theory and not a result of over-fitting, which can occur in using BR methods.

The multi-variant aspect of this research occurred in the evaluation of multiple models of varying data inputs for modeling the same predictive outputs. These were created in order to evaluate how structural and sociological characteristics relate to determine impact from wind related hazards. It was noted above that, among the PCIs, if the percent error was low, so were the false rates, while the true rates were higher (as desired). The percent error associated with an ANN is equivalent to how many data points fell within a network-produced output that did not match their target (known) output. In order to evaluate this, 10 ANNs of varying inputs were built as outlined in Supplementary Table S1 with Figure 2 (of the main text) relating how those variables fit in the ANN mathematical structure.

The 10 models were designed with the intent to learn what sociological factors interact best with hazard and structural related factors causally linking to impact, in the form of building damage state, from wind related events. It may also be possible that none of the subject factors interact well and this remains simply an engineering-related problem (Model 3). The results of building these 10 different models using BR, LM, and RP training are shown in Supplementary Figures S9 through S12. As can be seen, the best performing models include certain sociological factors such as housing tenure and per capita income. Additionally, the models with variables that relay debris potential to the ANN, such as percent area forested and housing density, performed very well. *What was also noticeable across each learning algorithm was that Model 3, solely hazard and engineering related inputs, was one of the poorer performing models, indicating that determining impact from wind hazards, in terms of physical damage state, is not solely a structural engineering-related problem*.


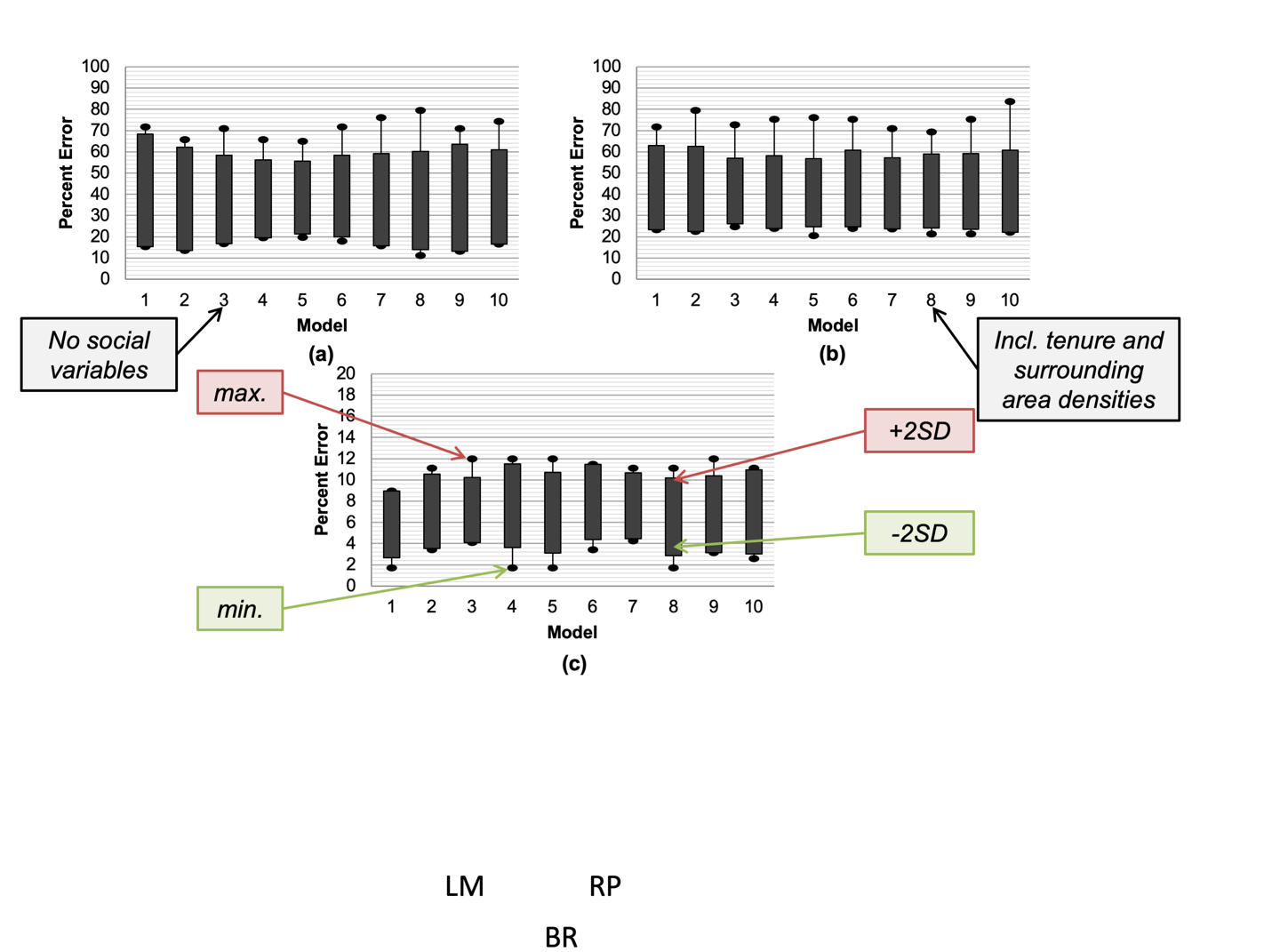


**Supplementary Figure S9.** Resulting percent error data ranges from 80+ ANN builds for varying damage models using (a) LM, (b) RP, and (c) BR training algorithms.


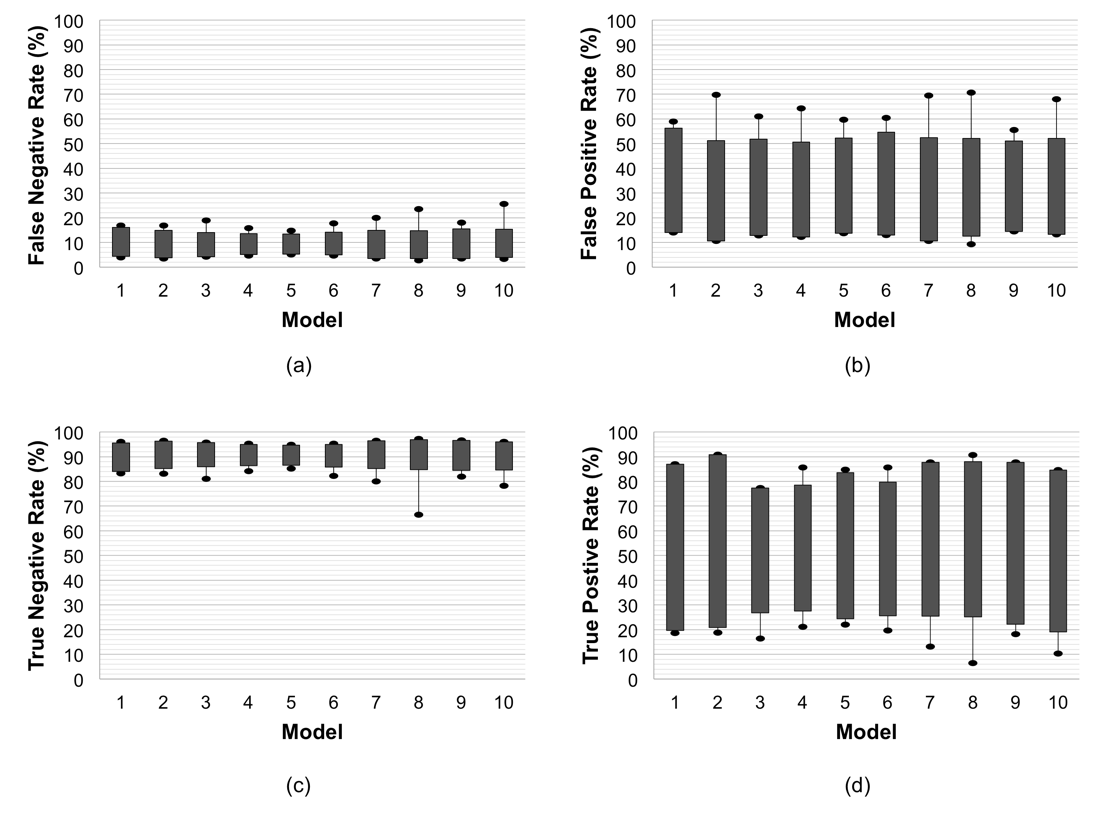


**Supplementary Figure S10.** Various Damage Models’ (a) FNR, (b) FPR, (c) TNR, and (d) TPR for LM.


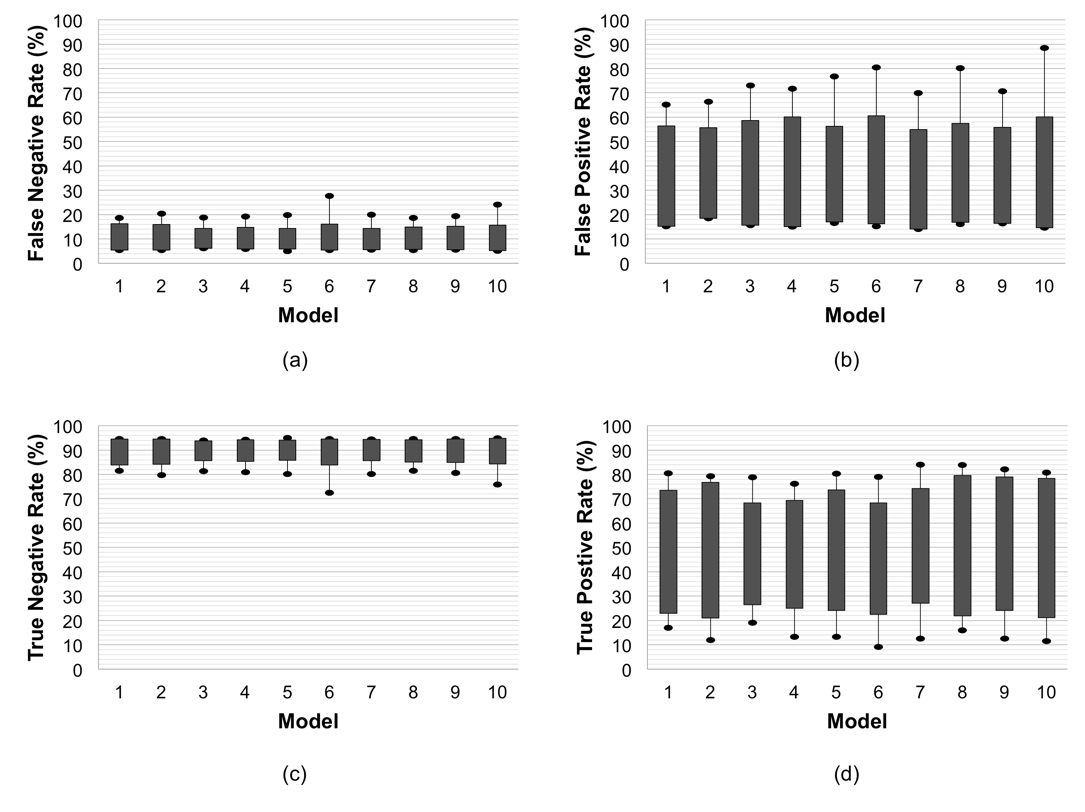


**Supplementary Figure S11.** Various Damage Models’ (a) FNR, (b) FPR, (c) TNR, and (d) TPR for RP.


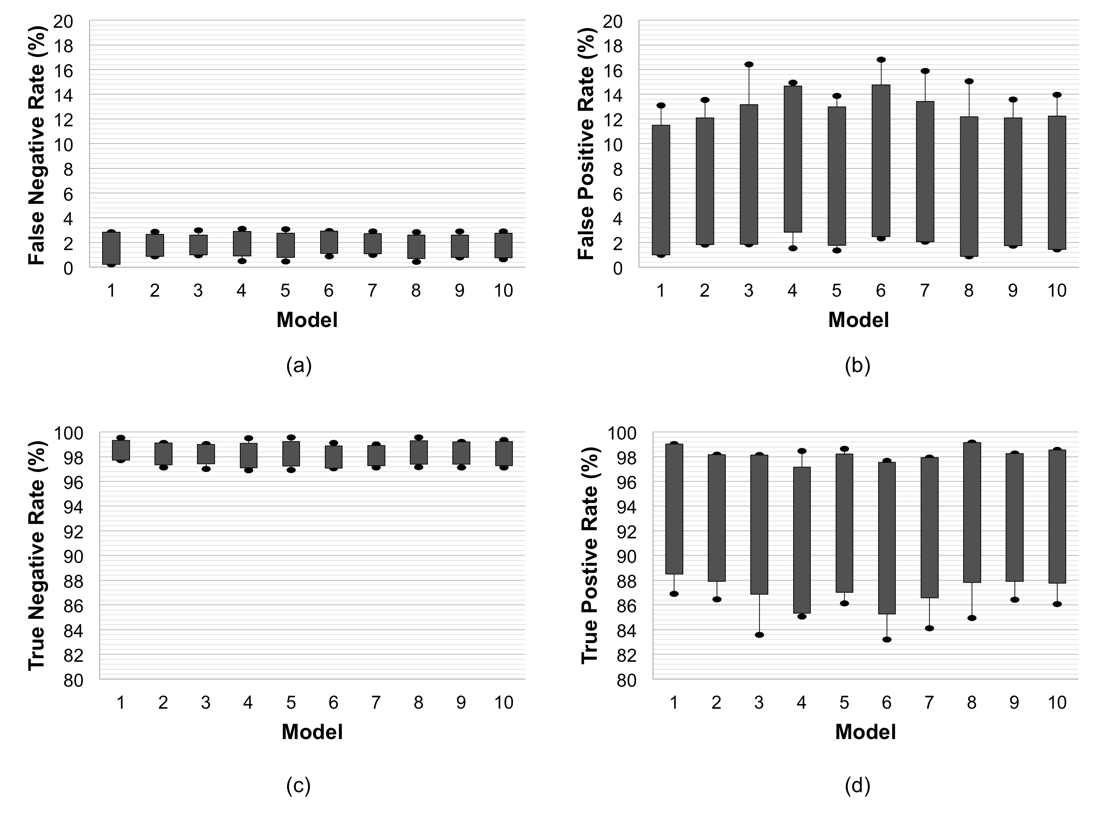


**Supplementary Figure S12.** Various Damage Models’ (a) FNR, (b) FPR, (c) TNR, and (d) TPR for BR.

The results from model variations with BR, LM, and RP algorithms, showed Damage Model 3 as one of the worst performing options and Damage Model 8 as one of the better performing options, indicating that the BR results showed a similar pattern in model comparison to that of the LM and RP results. Therefore, BR was determined viable for creating the final model ensemble consisting of multiple (designated A through F) ANNs.

As previously stated, Damage Model (DM) 3 served as a control comparison of how damage states have been historically modeled in considering the hazard and engineering characteristics (structural, surface roughness, wind speed). Damage Model (DM) 8 was built as a mix of DM5 and DM7 in consisting of the housing tenure (percent owners and percent renters) and the potential for debris impacts (percent forested area and the housing density) based on the relatively improved performance of these models across the three algorithm types. Both DM3 and DM8 had final model ensembles built for comparative evaluations in the following sections. Supplementary Figure S13 shows the percent error of the ensemble ANNs A through F for both DMs, as well as the confusion matrices for the lowest percent error ANNs of each model. The percent error of each ANN build is defined as the percent of data points incorrectly matched to their known target outcome during the training, validation, and testing phases. Within a confusion matrix, ANN outputs were compared to the desired targets. If the outputs matched the targets for a data point it was tallied along the diagonal of the matrix. The confusion matrices illustrated that many data points were correctly placed to their resulting output damage state during training and testing. The matrices shown in Supplementary Figure S13 are examples; it is possible to reach the same percent error with differing misplaced data points. These final ensemble ANNs all fall within the 50% lowest possible error from the build analysis, shown in Figure 4‑5, and with ROCs clustered primarily in the “true positive” region as shown in Supplementary Figure S14.


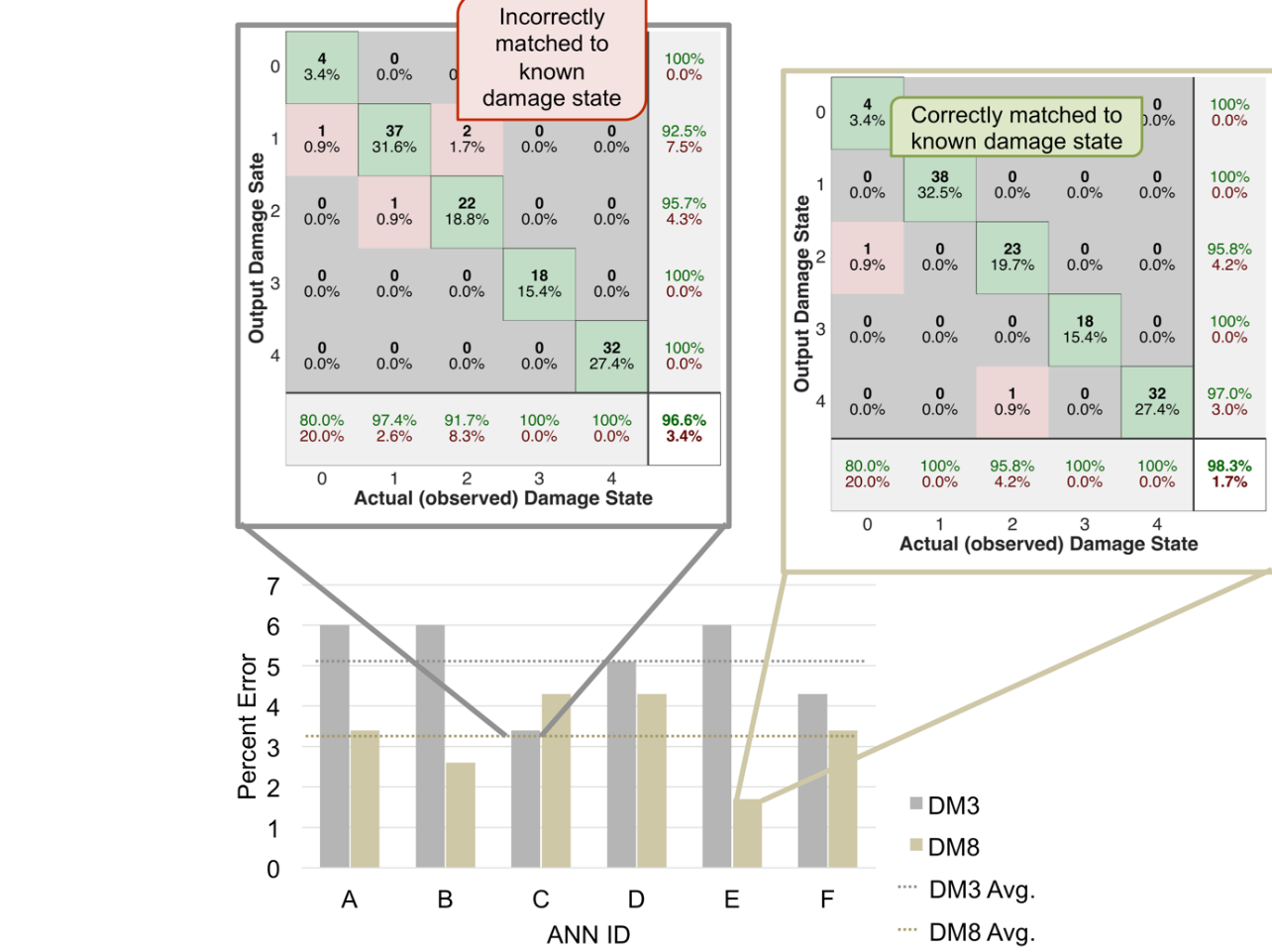


**Supplementary Figure S13.** Final Ensemble ANNs’ build percent error and how that error occurred through the training process as shown by respective confusion matrices.


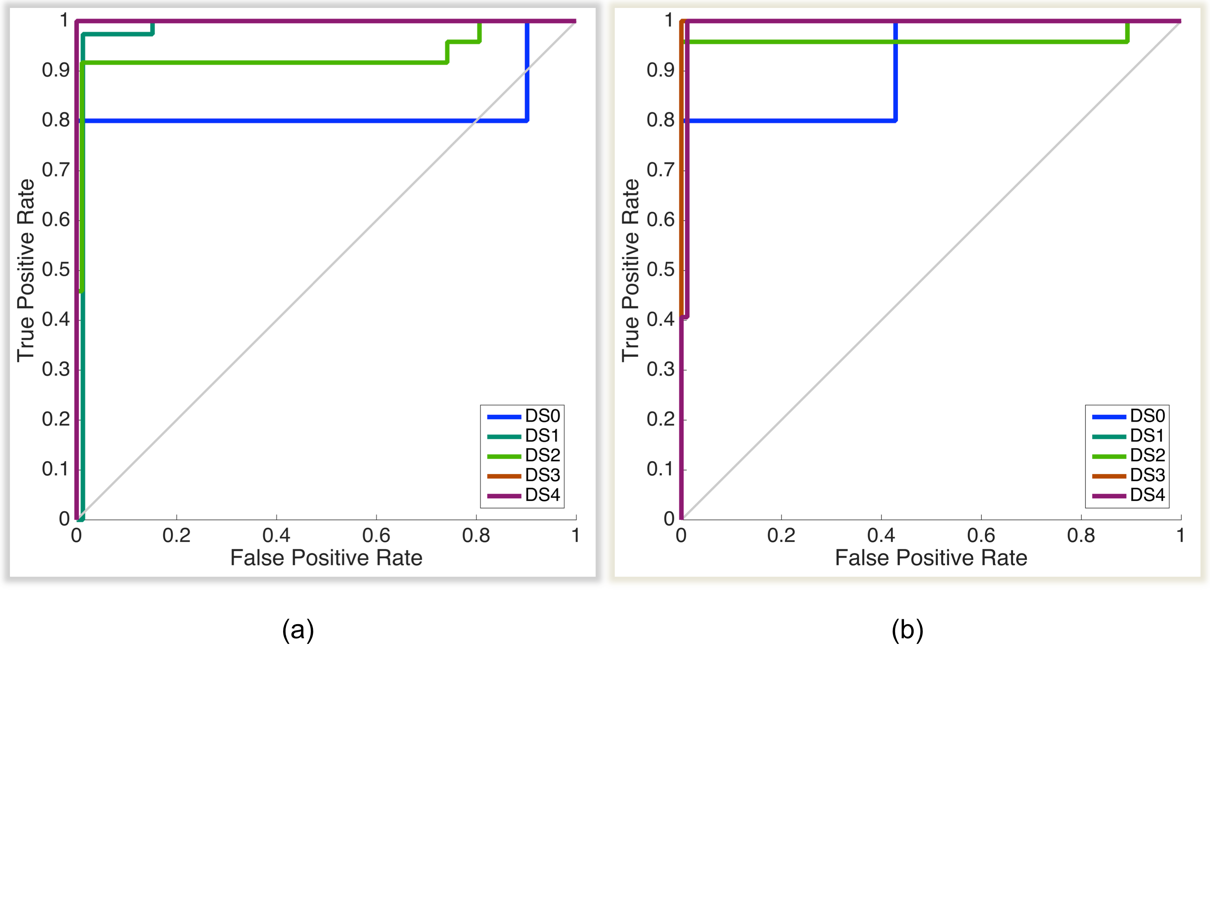


**Supplementary Figure S14.** ROCs for ensemble ANNs (a) DM3-C and (b) DM8-E.

Having a perfectly performing ANN is rather unrealistic, but a desirable ANN will have a lower number of incorrectly placed data points and primarily high true positive rates. DM3’s percent error was higher for all algorithm types with some having data points output as far off as three damage states. However, DM8 showed a focus in error in the DS3 and DS4 regions for those it did misplace. A 1.7% error (BR) was the lowest percent error reached across all algorithms and model builds.

From the above results and analysis, BR was considered the best performing training algorithm with DM8 being the best structure. The final machine learning model for predicting impact to a community’s building stock, therefore, consisted of an ensemble of 6 ANNs requiring DM8 inputs, with 10 hidden neurons, and 5 output damage states, trained using BR to reach a lowest possible MSE. These final 6 ANNs for DM3 and DM8 were used for and hindcasting individual building damage from the 2011 Joplin Tornado and for the graphical analysis within the main text as Models (A) and (B), respectively.

**Validation through Hindcasting the 2011 Joplin Tornado**

As part of additional validation of this modeling approach, DM3 and DM8 were simulated for the conditions of the 2011 Joplin Tornado and an exact building-to-building match was evaluated using video data from Kent State University immediately following the event. When each individual building’s predictive damage state was analyzed against its matched observed damage state from video cataloged images, it was found that DM3 and DM8 approaches produced overall similar results with a match percentage of roughly 40-45%, indicating similar modeling accuracy when categorizing a community’s building stock on the building by building level. The ANN DM8 did categorize 301 buildings, of the 3,283 buildings recorded in the video data, to the observed damage state that DM3 missed (Figure 3 of the main article). Similarly, DM3 matched 184 buildings DM8 failed to correctly categorize. Since DM8 performed marginally better than DM3, a generalized comparison of the total buildings to be categorized in each damage state was also assessed. The results of this, shown in Supplementary Table S7, demonstrate a drastic increase in accuracy when considering how many total buildings within the community were considered a total loss (DS3 and DS4) or not (DS1 and DS2), which may arguably be the more important distinction.

**Supplementary Table S7.** Total buildings damaged as recorded by Jasper County, MO and the United States Army Corps of Engineers (USACE) with comparison to physics-based fragilities and DM8 ANN results.

|  | Light (DS1) | Medium/  Moderate (DS2) | *Combined DS1 & DS2* | Totaled/  Extensive (DS3) | Demolished/  Catastrophic (DS4) | *Combined DS3 & DS4* | Total Buildings with Damage |
| --- | --- | --- | --- | --- | --- | --- | --- |
| Jasper County | 3,865 | 736 | *4,601* | 1,238 | 2,520 | *3,758* | 8,359 |
| USACE | 2,013 | 1,641 | *3,654* | 1,632 | 2,322 | *3,954* | 7,608 |
| Avg. |  |  | *4,127* |  |  | *3,856* | 7,983 |
| ANN DM8  Results | 2,519 | 1,074 | *3,593* ***(13% error)*** | 268 | 3,974 | *4,242* ***(10% error)*** | 7,835  **(2% diff)** |

Given the differences between survey data of different sources and the subjectivity of evaluating building damage state, these error values were considered acceptable. While the exact building to building match for DMs 3 and 8, along with the BR percent error, do not show much variation, the graphical analysis within the main text provided more insight into determining which model network structure best related the relevant variables.
